# *GDF6* Knockdown in a Family with Multiple Synostosis Syndrome and Speech Impairment

**DOI:** 10.3390/genes12091354

**Published:** 2021-08-29

**Authors:** Raymond A. Clarke, Zhiming Fang, Dedee Murrell, Tabrez Sheriff, Valsamma Eapen

**Affiliations:** 1 Ingham Institute, School of Psychiatry, University of NSW, Sydney, NSW 2170, Australia; z.fang@alumi.unsw.edu.au (Z.F.); v.eapen@unsw.edu.au (V.E.); 2 School of Medicine, St George Hospital, University of NSW, Sydney, NSW 2217, Australia; d.murrell@unsw.edu.au (D.M.); tabrez.sheriff@gmail.com (T.S.)

**Keywords:** multiple synostosis syndrome, vertebral fusion, GDF6, SYNS4, Klippel-Feil, progressive ossification, pisiform, skeletal morphology

## Abstract

Multiple synostoses syndrome type 4 (SYNS4; MIM 617898) is an autosomal dominant disorder characterized by carpal-tarsal coalition and otosclerosis-associated hearing loss. SYSN4 has been associated with GDF6 gain-of-function mutations. Here we report a five-generation SYNS4 family with a reduction in *GDF6* expression resulting from a chromosomal breakpoint 3′ of *GDF6*. A 30-year medical history of the family indicated bilateral carpal-tarsal coalition in ~50% of affected family members and acquired otosclerosis-associated hearing loss in females only, whereas vertebral fusion was present in all affected family members, most of whom were speech impaired. All vertebral fusions were acquired postnatally in progressive fashion from a very early age. Thinning across the 2nd cervical vertebral interspace (C2-3) in the proband during infancy progressed to block fusion across C2-7 and T3-7 later in life. Carpal-tarsal coalition and pisiform expansion were bilaterally symmetrical within, but varied greatly between, affected family members. This is the first report of SYNS4 in a family with reduced *GDF6* expression indicating a prenatal role for *GDF6* in regulating development of the joints of the carpals and tarsals, the pisiform, ears, larynx, mouth and face and an overlapping postnatal role in suppression of aberrant ossification and synostosis of the joints of the inner ear (otosclerosis), larynx and vertebrae. RNAseq gene expression analysis indicated >10 fold knockdown of *NOMO3*, *RBMXL1* and *NEIL2* in both primary fibroblast cultures and fresh white blood cells. Together these results provide greater insight into the role of GDF6 in skeletal joint development.

## 1. Introduction

Bone morphogenetic proteins (BMPs) regulate skeletal development, bone morphology and joint formation. Joints form when skeletal elements segment and develop articulations that provide flexibility, strength and versatility through specialised functions and appendages [1]. Much of what we now know regarding the molecular basis of skeletal joint development derives from our understanding of genetic changes affecting *BMP13* and *BMP14*, more commonly referred to as growth and differentiation factor 6 *(GDF6)* and 5 (*GDF5)*, respectively [1,2,3,4,5].

GDF6 joint phenotypes overlap GDF5 joint phenotypes, including carpal, tarsal and vertebral fusion [6,7,8]. GDF5 gain-of-function mutations increase the downstream signalling of GDF5 causing proximal symphalangism (SYM) and multiple synostoses syndrome type 2 (SYNS2; MIM 610017) which is characterised by the fusion of the carpals, tarsals and vertebrae [6,7,8]. By comparison, GDF5 loss-of-function mutations cause brachydactyly (BDA and BDC) [8,9]. GDF6 gain-of-function mutations cause multiple synostoses syndrome type 4 (SYNS4) [3,4,5] which is characterised by synostoses of the carpals and tarsals and otosclerosis-associated conductive hearing loss but not vertebral fusion [3,4,5]. Leber congenital amaurosis-17 (LCA17, MIM 615360) and isolated cases of microphthalmia (MCOP4, MIM 613094) have also been reported in association with *GDF6* loss-of-function mutations [10,11]. In contrast, increased levels of *GDF6* expression, which derive from the recurrent duplication of the *GDF6* gene in Leri’s pleonosteosis are characterised by vertebral fusion, reduced joint flexibility and mobility and flexion contractures of the interphalangeal joints [12]. 

In this study we report a SYNS4 family with classical carpal-tarsal coalition and acquired otosclerosis-related conductive hearing loss [3,4,5]. All affected family members presented with variable degrees of vertebral fusion and most were speech impaired. 

## 2. Materials and Methods

**Figure 1:**
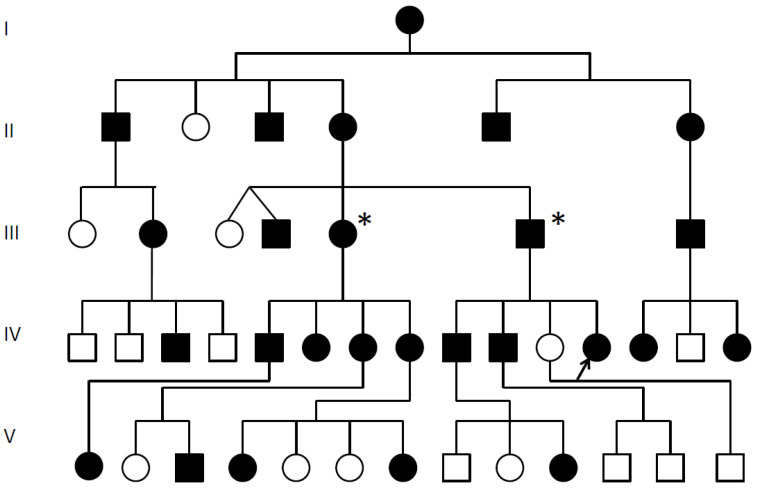
SYSN4 family pedigree. 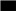 Multiple synostoses, vertebral fusion, speech impairment; chromosomal breakpoint 3′ of *GDF6*; * Decreased *GDF6* expression confirmed; Proband arrowed; 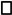 Unaffected.

Clinical Investigation: Radiology of the wrists, feet and spine were performed using routine practices [13]. We conducted a long-term review of radiographs and reports compiled over 30 years. We performed a detailed review of radiographs of the hands, feet and spine from four affected family members IV-5, IV-9, IV-10 and IV-12 (Figure 1 and Table 1). Other members of this family have been described previously with what was misdiagnosed as congenital vertebral fusion (Klippel-Feil anomaly; MIM 118100) [13]. Hearing tests and endoscopic examinations of the larynx and skeletal radiographs were performed at clinics across Australia. There were no reports of vision impairment or other ocular anomalies in this family. 

Primary cell culture: Primary fibroblasts acquired from skin biopsies were cultured in DMEM supplemented with 10% fetal calf serum (FCS). Cells were centrifuged at 1000 rpm for 5 min, resuspended and washed in PBS before RNA isolation and comparative rtPCR analysis as described elsewhere [14].

RNA isolation: Total RNA was extracted from fresh white blood cells and fibroblast cultures using Trizol according to the manufacturer’s instructions (Thermo Fisher Scientific, Sydney, Australia). Purelink RNA mini kit (12183025) spin columns(#12183025 Thermo Fisher Scientific, Sydney, Australia) were used for RNA purification. RNA was resuspended in DEPC-treated water and quality tested using a fragment Analyser (Agilant, Mulgrave, Australia) followed by the addition of 1 uL of RNASEOUT ((#10777019 Thermo Fisher Scientific, Sydney, Australia). 

Comparative rtPCR: First-strand cDNA synthesis was performed using the SuperScript™ III First-Strand synthesis rtPCR Kit (Invitrogen Cat# 11752-050, Thermo Fisher Scientific, Sydney, Australia) according to manufacturers’ instructions: 10 μL of 2 x RT Reaction Mix, 2 μL RT Enzyme Mix and 50 pg of purified RNA were made up to 20μL with DEPC-treated water and incubated at 25 °C for 10 min and again at 42 °C for 50 min. Reactions were terminated at 85 °C for 5 min, then chilled on ice for 5 min followed by a short spin in the microfuge. Then 1μL (2 U) of E. coli RNase H was added and incubated at 37 °C for 20 min. 

PCR master mix was prepared from a common stock reaction mix. Volumes for a 25 μL reaction were as follows: 12.5 μL of Platinum^®®^ SYBR^®®^ Green qPCR SuperMix-UDG (#11733-046 Thermo Fisher Scientific, Sydney, Australia.), 1 μL each of 10 μM primer stocks specific for the genes of interest (Table 2), 2.5μL of cDNA and DEPC-treated water to 25 μL. Reactions were incubated at 50 °C for 2 min and an initial denaturation step of 94 °C for 2 min. PCR was performed for 40 cycles with denaturation at 94 °C for 15 sec, annealing at 55 °C for 10 sec and extension at 72 °C for 20 sec. Comparative PCR profiles were independently normalised against the expression of two house-keeping genes (*GAPDH* and *18sRNA)* to remove any non-biological variation. 

mRNA sequencing by Illumina HiSeq /Novaseq: Total RNA of each sample was extracted using TRIzol Reagent and Purelink RNA mini kit columns. Total RNA of each sample was quantified and qualified by an Agilent 2100/2200 Bioanalyzer (Agilent Technologies, Palo Alto, CA, USA), NanoDrop (Thermo Fisher Scientific, Sydney, Australia). 1 μg total RNA was used for library preparation. Next generation sequencing library preparations were constructed according to the manufacturer’s protocol by Genewiz China. 

Differential expression analysis used the DESeq2 Bioconductor package, a model based on the negative binomial distribution. The estimates of dispersion and logarithmic fold changes incorporate data-driven prior distributions, Padj of genes were set <0.05 to detect differentially expressed genes. For expression analysis transcripts in Fasta format were converted from known gff annotation file and indexed properly. Then, with this file as a reference gene file, HTSeq (v0.6.1) estimated gene and isoform expression levels from the pair-end clean data. For GO and KEGG enrichment analysis GOSeq (v1.34.1) was used to identify Gene Ontology (GO) terms that annotate a list of enriched genes with a significant padj < 0.05. TopGO was used to plot DAG. Differentially expressed genes are presented in order of fold change relative to unaffected control.

## 3. Results

### 3.1. Radiological Review 

This is the first long-term review of a SYNS4 family. We review a 30-year history of radiological films and reports for the affected family (Figure 1) where ~50% of the affected displayed carpal and tarsal coalition, all affected family members displayed expansion of the pisiform and six of the seven affected females tested presented with otosclerosis-associated conductive hearing loss that was absent from affected males. All affected family members presented with variable degrees of vertebral fusion and most were speech impaired. 

#### 3.1.1. Female Proband (IV-12)

**Figure 2:**
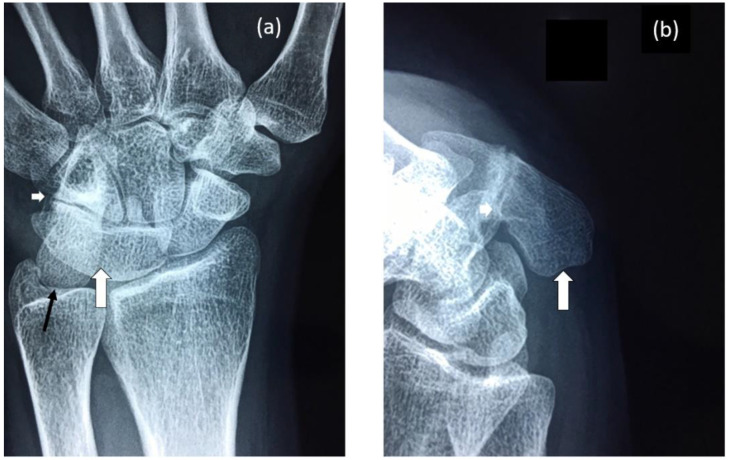
Radiographs of proband’s wrist highlighting: (**a**) Fusion of lunate and triquetrum (large white arrow), nearly complete fusion of hamate and pisiform (small white arrow) and elongation of the pisiform (black arrow). (**b**) Elongated pisiform (large white arrow) and fusion of hamate and pisiform (small white arrow).

**Figure 3:**
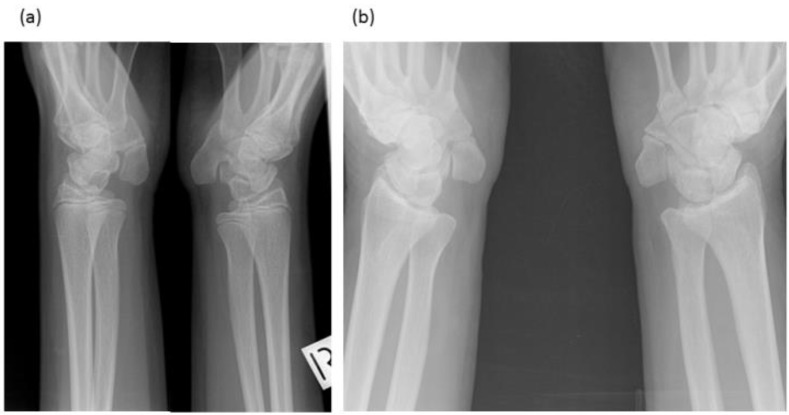
Radiographs of wrists of: (**a**) Proband—display bilateral symmetry of elongation of the pisiforms. (**b**) Proband’s father—displays bilateral symmetry of elongation of the pisiforms which are different in length and morphology from those of the proband. Both father and daughter had restricted wrist rotation/supination and grasping and writing capacity.

**Figure 4:**
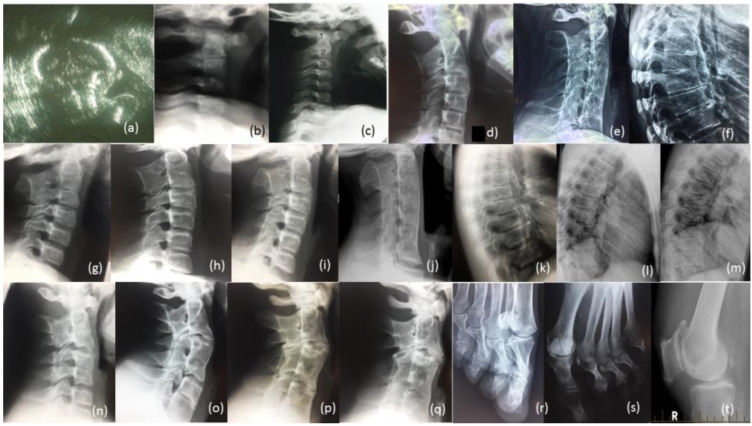
Radiological review of family. Legend: (**a**) Prenatal ultrasound of cervical spine of proband 36 Weeks; (**b**) Radiographs of cervical spine of proband age 10 weeks, (**c**) 12 months (**d**) 13 years (**e**) 27 years; (**f**) Radiograph of thoracic spine of proband age 27 years; (**g**) Radiograph of cervical spine of brother IV-10 age 7 years, (**h**) age 14 years, (**i**) age 17 years, (**j**) age 26 years; (**k**) Radiograph of thoracic spine of IV-10 age 17 years, (**l**) age 26 years, (**m**) father III-6; (**n**) Radiograph of cervical spine of brother IV-9 age 12 years, (**o**) age 19 years, (**p**) cousin IV-5 age 17 years, (**q**) age 24 years; (**r**) Bilateral deviation of the proximal phalanges cousin IV, (**s**) aunty III-2, (**t**) Bilateral spurs on patella Aunty III-5 age 65 years.

The female proband presented with bilateral carpal and tarsal coalition. In the feet there was bilateral fusion between the navicular and cuboid. In the hands there was bilateral fusion between the triquetrum and lunate and between the hamate and capitate and partial fusion between the hamate and pisiform (Figure 2). Bilateral elongation of the pisiforms was symmetrically similar in the proband (Figure 3a) and all other affected family members tested, but varied greatly in degree between affected family members (Figure 3b). Pisiform elongation associated with restricted wrist rotation/supination and grasping and capacity to write. Prenatal ultrasound of the proband provided no evidence of vertebral fusion (Figure 4a). Spinal radiographs at age 10 weeks for the proband confirmed the absence of congenital vertebral fusion in the cervical spine (Figure 4b). At age 12 months the proband had developed fusion of the C2-3 apophyseal joints and anterior regions of the spinous processes (Figure 4c). At age 13 there was complete fusion of the C2-3 vertebral bodies, fusion of the anterior edges of C3-4 and C4-5 vertebral bodies, progressive ossification of the anterior edges between C5-6 vertebral bodies, partial fusion of the apophyseal joints and spinous processes at C6-7 (Figure 4d) and partial fusion of T3-7 vertebral bodies. At age 27 spinal fusion of the vertebrae had progressed to form block fusion across C2-7 (Figure 4e) and a continuous set of partial fusions of the anterior edges of the vertebral bodies between T3-T7 (Figure 4f). The bilaterally elongated pisiforms of the proband did not increase in length or size over time nor did the fusion of carpals or tarsals appear to progress. Pisiform elongation was associated with restricted flexion and supination movement of the wrist. The proband presented with stiffness in the Achilles tendon with associated toe walking. She was the only female (age 27) tested that was negative for hearing loss and she was dysphonic from birth and displayed severe speech impairment which was associated with malformation of laryngeal cartilages in her speech-impaired father. Short tongue and microstomia was evident in the proband and most other affected family members in association with overcrowding of the teeth that required teeth removal.

#### 3.1.2. Brother (IV-10)

The youngest brother of the proband presented with bilateral fusion between the hamate and capitate and between the hamate and pisiform, but not between the lunate and triquetrum as observed in both the proband and her affected father. In addition, there was bilateral extension of the pisiform proximally that restricted supination of the wrists and apposition of the thumbs. There was bilateral fusion of the tarsals including cuneiform coalition and talocalcaneal coalition. Radiographs of the spine indicated progressive postnatal cranio-caudal acquisition of vertebral fusions beginning from C2-3. At age 7 this boy presented with fusion of the apophyseal joints and spinous processes of the 2nd and 3rd cervical (C2-3) vertebrae (Figure 4g) which by age 14 had progressed to complete fusion of the C2-3 vertebral bodies and thinning of the vertebral interspace at C3-4 (Figure 4h). At age 17 ossification of intervertebral disc spaces had progressed to where there was thinning of the C3-7 vertebral interspaces and fusion of the apophyseal joints at C2-6 on the right and C6-7 on the left (Figure 4i) which by age 26 had progressed to complete block fusion of C2-7 (Figure 4j). At age 26 he presented with vertebral fusion in the upper thoracic spine where there had only been thinning of these interspaces 9 years earlier (Figure 4k). At age 26 there was partial anterior fusion of the vertebral bodies at T4-T6 (Figure 4l) comparable to the thoracic fusion profile in the spine of his affected father III-6 (Figure 4m). Aged 7 years, he presented with very mild dysphonia, habitual toe walking, severely restricted flexion and supination movement in the hands, microtia, low set ears, short tongue, microstomia and overcrowding of the teeth that required teeth removal. Mobility of other joints appeared normal and there was no conductive hearing loss evident in this man or any other affected male member of the family.

#### 3.1.3. Brother (IV-9)

The proband’s oldest brother (IV-9) presented with postnatal fusion in the cervical spine which progressed in a cranio-caudal direction from C2-3. A solitary C2-3 fusion identified at age 12 (Figure 4n) had progressed 7 years later to include a C4-5 fusion and thinning of the C6-7 interspace (Figure 4o). There was no evidence of conductive hearing loss in this brother or any other affected male members of the family. He suffered severe dysphonia from birth associated with severe speech impairment, microstomia and overcrowding of the teeth.

#### 3.1.4. Male Cousin (IV-5)

The proband’s male cousin (IV-5) presented with progressive postnatal fusion of the cervical spine. Fusion of the C2-3, C4-5 and C6-7 cervical vertebrae at age 17 (Figure 4p) had progressed to C2-7 block fusion at age 24 (Figure 4q). At age 50 he experienced severe stiffness of the neck, shoulders and back. There was no evidence of conductive hearing loss. Severe speech impairment was evident from an early age. Short tongue and microstomia was associated with overcrowding of the teeth that required removal of eight teeth from him and all three of his affected sisters (IV-6, -7 and -8).

#### 3.1.5. Familial Skeletal Anomalies

Near 50% of affected family members tested presented with variable degrees of carpal and tarsal coalition. Carpal and tarsal fusions were bilaterally similar in extent and morphology within affected family members but varied between affected family members. Likewise, expansion of the pisiform was bilaterally similar in extent and appearance within affected family members but varied between affected family members. A total of six out of the seven affected female family members tested for hearing loss were diagnosed with otosclerosis and/or unilateral conductive hearing loss, including the proband’s grandmother (II-4) age 45, aunty (III-3) age 40, aunty (III-5) age 36, female cousin (IV-6) age 14, female cousin (IV-8) age 20 and niece (V-1) age 5, with evidence of deterioration with age. The only female tested that was negative for hearing loss was the proband, age 27 years (Figure 1).

All four of the affected family members reviewed here in detail (Table 1) presented with progressive postnatal acquisition of vertebral fusions (Figure 4). Ultrasound before birth for two affected family members (IV-12 and V-3) confirmed the absence of congenital vertebral fusion which later developed postnatally through progressive ossification. Vertebral fusion, carpal and tarsal coalition, bilateral pisiform elongation and vocal impairment were all variable in extent between affected family members. Bilateral deviation of the proximal phalanges of toes 2–5 was evident in two affected female members of the family (IV-8, see Figure 4r) and (III-2, see Figure 4s). The former female’s mother (III-5) developed painful age-related bilateral spurs on the patella at age 65 (Figure 4t) and age-related conductive hearing impairment age 40. Other skeletal anomalies included Perthes of the hip in one teenage male in association with a minimal fusion bridge between the C2-3 vertebrae and no obvious speech impairment (V-3). There was no evidence of restriction of the elbows or the shoulders in affected family members. Most affected family members presented with varying degrees of speech impairment from a young age in association with malformation of laryngeal cartilages including flattening of the anterior commissure and shortening of the vocal cords [15]. Surgical intervention in one affected family member reported that the vocal cords were shorter, failed to meet in the midline, were of a different complexion/composition and did not vibrate normally and that other vocal ligaments were ossified [15]. Short tongue and microstomia with overcrowding of the teeth were common. In more severely speech-affected members of the family there were deficits in verbal fluency and significant difficulty in generating words beginning with a certain letter [15]. Stature and intelligence appeared within the normal limits for all affected family members. Speech and hearing impairment affected learning; pisiform expansion restricted wrist rotation supination and grasping and capacity to write; narrowing of the oesophageal and laryngeal canals restricted swallowing and anaesthetic intubation; Achilles tendon stiffness was associated with toe walking; and arthritis progressed with age in many of the affected family members.

### 3.2. Gene Expression Analyses

#### 3.2.1. Comparative rtPCR Gene Expression Analysis

**Figure 5:**
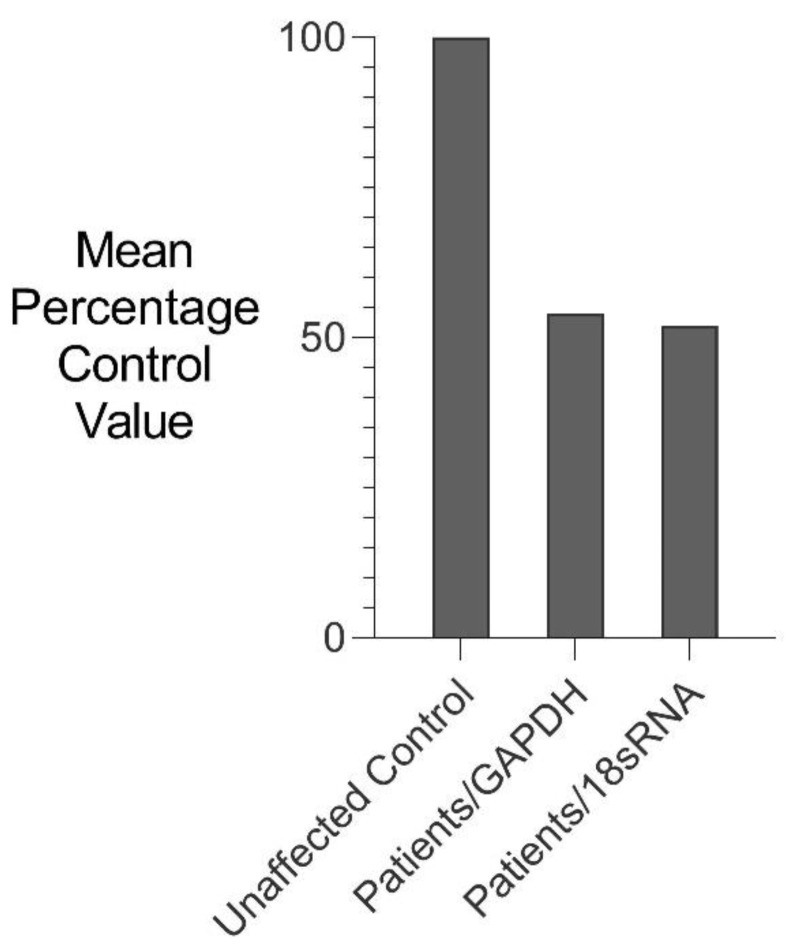
Comparative rtPCR analysis of *GDF6* expression in SYNS4 family. *GDF6* expression levels in fresh white blood cells expressed as the mean percentage change for two affected male members of the family compared with the mean for five age- and gender-matched unaffected controls independently normalised against the expression of two housekeeping reference genes *GAPDH* and *18sRNA*.

RNA isolated from fresh white blood cells derived from two severely affected family members (III-6 and IV-10) and five unaffected control individuals were analysed for changes in *GDF6* expression using comparative rtPCR expression analysis (Figure 5). *GDF6* expression levels were reduced in both affected family members when compared to the mean expression level for the five unaffected control individuals (Figure 5).

#### 3.2.2. RNA Sequencing Gene Expression Analysis (RNAseq)

RNA isolated from a severely affected family member with reduced *GDF6* expression (III-6) was used for RNAseq gene expression analysis. RNAseq analysis identified 68 and 61 genes with >10 fold differential gene expression in primary fibroblast cultures and fresh white blood cells, respectively (*p* < 0.05) (Table 3). Of these, 3 genes (*NOMO3*, *RBMXL1* and *NEIL2*) exhibited >10 fold knockdown in both primary fibroblasts and white blood cells from the affected family member compared with an age, gender and racially matched unaffected control individual (Table 3).

## 4. Discussion

In this study we report the first SYNS4 family with reduced *GDF6* expression. The three previously reported SYNS4 families had GDF6 gain-of-function mutations [3,4,5]. The family phenotype included the classical bilateral carpal and tarsal coalition and otosclerosis associated conductive hearing loss typical of SYSN4 [3,4,5]. In addition, there was progressive postnatal acquisition of vertebral fusions in the cervical and thoracic spine from an early age. The extent of the vertebral fusion was variable between affected family members; notwithstanding, all affected family members displayed some degree of fusion across the C2-3 vertebral interspace at the cranial end of the cervical spine. Most affected family members were also speech impaired in association with malformation of the laryngeal cartilages and what appeared to be progressive ossification of laryngeal ligaments and joints.

The decrease in *GDF6* expression in this family was associated with a chromosomal breakpoint 3′ of the *GDF6* gene [13]. *GDF6* encodes a bone morphogenetic protein which functions in a dose-dependent fashion in its extracellular regulation of skeletal development [1,16]. In consequence, the varying degrees of joint ossification and skeletal deformation in this family likely reflect variations in the reduction of *GDF6* dose at different skeletal sites in different affected family members, possibly due to position effects on *GDF6* regulatory elements located near the chromosomal breakpoint. Indeed, a conserved long-range *GDF6* pharyngeal specific enhancer (ECR5) that functions from the earliest stages of pharyngeal and otolaryngeal development has been located between *GDF6* and the chromosomal breakpoint in this family [1,17].

Otosclerosis-associated conductive hearing loss represents one of the classical acquired characteristics of SYSN4 [3,4,5]. In this family, otosclerotic hearing loss presented at varying ages (5–40 years of age) in affected females only, but not in affected males. This finding would appear to indicate a gender effect on the progressive ossification of the ossicles in this family. In contrast, speech impairment in this family was associated with congenital malformation and aberrant postnatal ossification of the larynx which was more severe in males compared to females and further exacerbated during descent of the thyroid cartilage during male puberty. Notwithstanding, the one affected male member of the family without speech impairment (V-3) also displayed the least degree of vertebral fusion.

*Gdf6* is expressed in discrete patterns within the developing joints of the mouse [1,2]. These same joints correspond precisely with those joints that were fused and malformed in the affected family including the joints of the carpals, tarsals, vertebrae, ear and larynx [1,2]. Moreover, knockout of *Gdf6* in mice results in the fusion of the carpals and tarsals but not the vertebrae [1]. This study identifies the biological basis of vertebral fusion in this family as aberrant progressive postnatal ossification and synostosis of the spinal joints from an early age. This mechanism is comparable and consistent with the aberrant postnatal ossification within the inner ear causing otosclerosis in SYNS4 [3,4,5]. In contrast, the precise mechanism of the carpal and tarsal fusions in this family remains uncertain. Notwithstanding, the bilateral symmetry of the carpal, tarsal and pisiform fusions/malformations within affected family members appears more consistent with a prenatal error of development, possibly effecting aberrant and excessive bone condensation/ossification prenatally. This fusion scenario is supported by animal studies which indicate a role for Gdf6 in stimulating chondrogenesis at early stages of development [16] while in vitro studies indicate that GDF6 has a distinct inhibitory effect on ossification and mineralization at later-stage, differentiated chondrocytes or osteoblasts [18]. GDF6 is a bone morphogenetic protein that acts extracellularly as a morphogen during development. Albeit the pathway down stream of GDF6 in determining cell fate and function has not been fully elucidated [3,4,5]. For insight into this pathway we searched for changes in gene expression in a severely affected family member (III-6) with reduced *GDF6* expression (Figure 5 and Table 3). RNAseq expression analysis identified >10 fold knockdown of three genes, *NOMO3*, *RBMXL1* and *NEIL2*, in both primary fibroblast cultures and fresh white blood cells from this severely affected family member (Table 3) [19,20,21,22]. This limited but close correlation in differential gene expression between disparate cell types provides a high level of confidence with respect to ongoing pathway and gene therapy investigations, not only in these three genes but the other genes differentially expressed in this patient (Table 3). It is therefore likely that one or more of these down-regulated genes has a role in the GDF6 pathway to skeletal joint development and ossification. RBMXL1 is of particular interest as it strengthens DNA heterochromatin binding, impedes the activity of transcription factors, suppresses gene transcription and serves as a barrier to direct cell conversion. Knockdown of RBMXL1 increases gene transcription [19]. NOMO3 is another molecule of interest as it is a positive modulator of the morphogen Nodal which is down regulated >5fold in patients with facial asymmetry and jaw malformations. Nodal is a transcription factor regulated by asymmetric cascades of morphogens. Nodal initiates the molecular pathway that induces chirality and asymmetry in endoderm and mesoderm germ layers during late gastrulation and neurulation [20,23].

## 5. Conclusions

Prenatal ultrasound followed with long-term radiological evaluation of the skeleton helped to differentiate between congenital, acquired, postnatal and progressive skeletal ossification and associated skeletal malformations. This study indicates a role for *GDF6* in the prenatal development of the joints of the hands and feet and larynx and in the progressive postnatal ossification of the joints of the inner ear, larynx and vertebral column. These findings further suggest that all *GDF6*-associated vertebral fusions may result from aberrant postnatal ossification, including those vertebral fusions previously assumed but never proven to be congenital errors of segmentation [10,13].

## Figures and Tables

**Table 1:** Clinical details of selected family members.

Family Member	Sex	Ages	Anomalies
Proband (IV-12)	F	0–27	Carpal Tarsal Coalition, Pisiform elongated No hearing impairment or congenital vertebral fusion, Postnatal vertebral fusion, speech impaired, short tongue and microstomia.
Brother (IV-10)	M	7–26	Carpal Tarsal Coalition, Pisiform elongated No hearing impairment or congenital vertebral fusion, Postnatal vertebral fusion, No speech impairment, short tongue and microstomia.
Brother (IV-9)	M	12–19	Carpal Tarsal Coalition, Pisiform elongated No hearing impairment, Postnatal vertebral fusion, Severe speech impairment, short tongue and microstomia.
Cousin (IV-5)	M	17–50	Carpal Tarsal Coalition, Pisiform not tested No hearing impairment, Progressive vertebral fusion, Severe speech impairment, short tongue and microstomia.

**Table 2:** PCR Primers.

Primer Direction	Primer Sequence
*GDF6*—forward	CCTGTTGCTTGTTTGGTTCA
*GDF6*—reverse	GCTGTCCATTTCCTCTTTGC
*18S rRNA*—forward	GTAACCCGTTGAACCCCATT
*18S rRNA*—reverse	CCATCCAATCGGTAGTAGCG
*GAPDH*—forward	CCACCCATGGCAAATTCCATGGCA
*GAPDH*—reverse	TCTAGACGGCAGGTCAGGTCCACC
Stealth control—sense	CAAGAACAGCGAGAAGCAGCCGUCA
Stealth control—antisense	UGACGGCUGCUUCUCGCUGUUCUUG

**Table 3:** Differentially expressed genes in RNA sequence analysis.

Fibroblast Cell Lines				White Blood Cells			
Down-Regulation		Up-Regulation		Down-Regulation		Up-Regulation	
Change	Gene	Change	Gene	Change	Gene	Change	Gene
−15.3854 −15.0439 −14.9457 −14.7973 −14.7586 −14.6305 −14.5996 −14.4498 −14.1642 −14.0789 −14.0487 −14.0126 −13.7795 −13.7398 −13.6138 −13.3585 −13.2922 −13.2741 −13.2696 −13.2425 −13.2212 −13.1889 −13.1802 −13.104 −12.7709 −12.6132 −11.8528 −11.8236 −11.801	NOMO3 * DNAH1 PRDM10 IDO1 SP140L MYSM1 GRB10 PTGS2BLOC1S2 TMEM260 SNX13 RCBTB1 CCP110 RBMXL1 * EPHX2 NF2 GZF1 CDCA8 AMZ2 POLR1B TTI1 WDR19 ABCB7 SMC6 DERA ZNF33B POLR3B NEIL2 * BEGAIN	10.4092 11.4755 11.9372 12.1726 12.301 12.4321 12.5031 12.5352 12.7603 12.833 12.9472 13.1595 13.1675 13.2051 13.2154 13.2714 13.276 13.3216 13.3411 13.7474 13.7502 14.072 14.0828 14.1284 14.1698 14.341 14.5617 14.7657 14.7712 14.8012 15.2457 15.7457 16.0123	MDGA1 GMEB1 CSTF2 RPGRIP1 TRIT1 ADGRA2 PCGF1 GAL3ST4 POM121 HTATIP2 PHETA1 MAP2K6 AP5S1 TBP KIAA023 2 ZNF550 SOCS2 ZNF280D EML3 FECH GZF1 ZNF331 DAG1 MKS1 AKAP7 COG6 ZNF302 PAX5 ZNF628 ZNF133 POT1 ZNF57 CACNB3	−15.077 −15.0329 −14.6255 −14.4338 −14.2377 −14.0202 −13.9483 −13.9278 −13.7308 −13.6908 −13.6735 −13.2039 −12.9068 −12.7789 −12.676 −12.624 −12.6236 −12.603 −12.5258 −12.4878 −12.4472 −12.1771 −12.1662 −12.0355 −11.5972 −11.497 −11.2932 −11.0185 −10.4318	AK4 GPR68 CDK19 PHETA1 ABHD16B ZNF229 TSC1 BTBD3 TMEM134 AFMID LIG1 GRAMD1A TRIT1 ZBTB43 MTR NOMO3 * RBMXL1 * RPF2 SP100 KRBA1 MORC2 UBE2F LRRN3 MAP3K4 NEIL2 * EPHA4 NT5M REEP2 ZFYVE27	11.0154 11.1429 11.8143 11.8187 12.0527 12.1243 12.1532 12.1681 12.3948 12.4649 12.6268 12.6402 12.8589 13.0328 13.0354 13.0485 13.0628 13.086 13.1499 13.1582 13.2509 13.2561 13.3067 13.3068 13.3209 13.4586 13.617 13.6751 13.8247 13.8448 13.9705 14.1731 14.3364 14.5402 14.8302 14.9429 15.0595 15.5187 15.8286 16.3832	TMEM201 ARNTL SLC25A38 ADARB1 ZCCHC4 BBS5 UCP2 ATP6V0A1 CEP83 NMNAT1 TMEM209 IQCE PHTF1 UBL4A GOLPH3L MED19 GLI2 XKR8 ZBED8 SCML1 NEDD9 ROR1 ZC3H8 CEP44 ZNF274 POGZ TENT5A TNIK TOP3A BTRC INPP4A PIK3R1 ELAPOR2 NAV3 GALNT15 NUP155 ADGRB2 SLC4A3 MPDZ ZNF510

* Genes downregulated in both fibroblast cell lines and fresh white blood cells.

## Data Availability

please refer to suggested Data Availability Statements in section “MDPI Research Data Policies” at https://www.mdpi.com/ethics.

## References

[1] Settle S.H., Rountree R.B., Sinha A., Thacker A., Higgins K., Kingsley D.M. (2003). Multiple joint and skeletal patterning defects caused by single and double mutations in the mouse Gdf6 and Gdf5 genes. Dev. Biol..

[2] Mortlock D.P., Guenther C., Kingsley D.M. (2003). A General Approach for Identifying Distant Regulatory Elements Applied to the Gdf6 Gene. Genome Res..

[3] Wang J., Yu T., Wang Z., Ohte S., Yao R.-E., Zheng Z., Geng J., Cai H., Ge Y., Li Y. (2016). A New Subtype of Multiple Synostoses Syndrome Is Caused by a Mutation inGDF6That Decreases Its Sensitivity to Noggin and Enhances Its Potency as a BMP Signal. J. Bone Miner. Res..

[4] Terhal P.A., Verbeek N.E., Knoers N., Nievelstein R.J.A.J., Ouweland A.V.D., Sakkers R.J., Speleman L., Van Haaften G. (2018). Further delineation of the GDF6 related multiple synostoses syndrome. Am. J. Med. Genet. Part A.

[5] Berentsen R.D., Haukanes B.I., Júlíusson P.B., Rosendahl K., Houge G. (2018). A Novel GDF6 Mutation in a Family with Multiple Synostoses Syndrome without Hearing Loss. Mol. Syndr..

[6] Schwaerzer G.K., Hiepen C., Schrewe H., Nickel J., Ploeger F., Sebald W., Mueller T., Knaus P. (2011). New insights into the molecular mechanism of multiple synostoses syndrome (SYNS): Mutation within the GDF5 knuckle epitope causes noggin-resistance. J. Bone Miner. Res..

[7] Dawson K., Seeman P., Sebald E., King L., Edwards M., Williams J., Mundlos S., Krakow D. (2006). GDF5 Is a Second Locus for Multiple-Synostosis Syndrome. Am. J. Hum. Genet..

[8] Seemann P., Brehm A., König J., Reissner C., Stricker S., Kuss P., Haupt J., Renninger S., Nickel J., Sebald W. (2009). Mutations in GDF5 Reveal a Key Residue Mediating BMP Inhibition by NOGGIN. PLoS Genet..

[9] Yang W., Cao L., Liu W., Jiang L., Sun M., Zhang D., Wang S., Lo W.H.Y., Luo Y., Zhang X. (2008). Novel point mutations in GDF5 associated with two distinct limb malformations in Chinese: Brachydactyly type C and proximal symphalangism. J. Hum. Genet..

[10] Asai-Coakwell M., French C.R., Ye M., Garcha K., Bigot K., Perera A.G., Staehling-Hampton K., Mema S., Chanda B., Mushegian A. (2009). Incomplete penetrance and phenotypic variability characterize Gdf6-attributable oculo-skeletal phenotypes. Hum. Mol. Genet..

[11] Ye M., Berry-Wynne K.M., Asai-Coakwell M., Sundaresan P., Footz T., French C.R., Abitbol M., Fleisch V.C., Corbett N., Allison T. (2009). Mutation of the bone morphogenetic protein GDF3 causes ocular and skeletal anomalies. Hum. Mol. Genet..

[12] Banka S., Cain S.A., Carim S., Daly S.B., Urquhart J., Erdem G., Harris J., Bottomley M., Donnai D., Kerr B. (2014). Leri’s pleonosteosis, a congenital rheumatic disease, results from microduplication at 8q22.1 encompassing GDF6 and SDC2 and provides insight into systemic sclerosis pathogenesis. Ann. Rheum. Dis..

[13] Clarke R., Singh S., McKenzie H., Kearsley J.H., Yip M.Y. (1995). Familial Klippel-Feil syndrome and paracentric inversion inv(8)(q22.2q23.3). Am. J. Hum. Genet..

[14] Fang Z., Eapen V., Clarke R.A. (2017). CTNNA3 discordant regulation of nested LRRTM3, implications for autism spectrum disorder and Tourette syndrome. Meta Gene.

[15] Clarke R.A., Davis P.J., Tonkin J. (1994). Klippel-Feil Syndrome Associated with Malformed Larynx. Case report. Ann. Otol. Rhinol. Laryngol..

[16] Pregizer S., Mortlock D.P. (2009). Control of BMP gene expression by long-range regulatory elements. Cytokine Growth Factor Rev..

[17] Reed N.P., Mortlock D.P. (2010). Identification of a distant cis-regulatory element controlling pharyngeal arch-specific expression of zebrafish gdf6a/radar. Dev. Dyn..

[18] Shen B., Bhargav D., Wei A., A Williams L., Tao H., Ma D.D.F., Diwan A. (2009). BMP-13 Emerges as a Potential Inhibitor of Bone Formation. Int. J. Biol. Sci..

[19] Becker J., McCarthy R.L., Sidoli S., Donahue G., Kaeding K., He Z., Lin S., Garcia B.A., Zaret K.S. (2017). Genomic and Proteomic Resolution of Heterochromatin and Its Restriction of Alternate Fate Genes. Mol. Cell.

[20] Nicot R., Hottenstein M., Raoul G., Ferri J., Horton M., Tobias J.W., Barton E., Gelé P., Sciote J.J. (2014). Nodal Pathway Genes Are Down-regulated in Facial Asymmetry. J. Craniofacial Surg..

[21] Scheffler K., Jalland C.M., Benestad S.L., Moldal T., Ersdal C., Gunnes G., Suganthan R., Bjørås M., Tranulis M.A. (2020). DNA glycosylase Neil2 contributes to genomic responses in the spleen during clinical prion disease. Free. Radic. Biol. Med..

[22] Sarker A.H., Chatterjee A., Williams M., Lin S., Havel C., Iii P.J., Boldogh I., Hazra T.K., Talbot P., Hang B. (2014). NEIL2 Protects against Oxidative DNA Damage Induced by Sidestream Smoke in Human Cells. PLoS ONE.

[23] Vandenberg L.N., Levin M. (2013). A unified model for left–right asymmetry? Comparison and synthesis of molecular models of embryonic laterality. Dev. Biol..

